# Ultrasound-guided percutaneous transhepatic biliary drainage for distal biliary malignant obstructive jaundice

**DOI:** 10.1038/s41598-024-63424-x

**Published:** 2024-05-30

**Authors:** Qingyuan Cai, Xiaomin Wu

**Affiliations:** Department of Ultrasound, Quanzhou First Hospital Affiliated to Fujian Medical University, Quanzhou, China

**Keywords:** Percutaneous transhepatic biliary drainage, Malignant obstructive jaundice, Ultrasound, Intervention, Tumour, Pancreatic cancer, Outcomes research

## Abstract

The main cause of distal biliary malignant obstructive jaundice (DBMOJ) is the stricture of the extrahepatic biliary tract by malignant tumors, including pancreatic head and uncinate process cancer, low-grade cholangiocarcinoma, duodenal cancer, papillary duodenal cancer and other malignant tumors. The most effective treatment is radical pancreaticoduodenectomy. However, preoperative obstructive jaundice can affect the patient’s liver function and blood coagulation function, increase local inflammation and oedema, and make surgery more difficult. Patients with severe obstructive jaundice require preoperative biliary drainage, which can be achieved by various methods, including ultrasound endoscopic biliary drainage (EUS-EBD) and endoscopic retrograde biliary drainage (ERBD). The latter is mainly divided into endoscopic nasobiliary drainage and endoscopic biliary stent. Some patients underwent percutaneous transhepatic biliary drainage (PTBD) when ERBD and EUS-EBD failed. In this study, we aimed to identify PTBD in DBMOJ and to further investigate the role of the puncture pathway in DBMOJ. The relationship between PTBD and bile duct internal diameter was confirmed by analysing and collating clinical data. In this study, DBMOJ was grouped according to bile duct internal diameter and liver function was used as an indicator to examine the improvement in liver function with PTBD in patients undergoing DBMOJ. Analysis of puncture complications showed that PTBD puncture was safe. DBMOJ with different bile duct internal diameters had different rates of liver function improvement after PTBD. The right-side approaches had significantly lower alanine aminotransferase (ALT) and alanine transaminase (AST) than the left-side approaches. This study showed that PTBD for DBMOJ is associated with a low complication rate and good reduction of jaundice. Liver function recovery was faster in patients with DBMOJ treated with PTBD in the right-sided approach compared with the left-sided approach. PTBD is an effective tool to be used in patients who have failed ERBD and EUS-EBD.

## Introduction

The main cause of distal biliary malignant obstructive jaundice (DBMOJ) is the stricture of the extrahepatic biliary tract by malignant tumors, including pancreatic head and uncinate process cancer, low-grade cholangiocarcinoma, duodenal cancer, papillary duodenal cancer and other malignant tumors. The most effective treatment is radical pancreaticoduodenectomy, which is often supplemented by postoperative radiotherapy, chemotherapy, and targeted therapy^1^. However, preoperative obstructive jaundice can affect the patient’s liver function and blood coagulation function, aggravate local inflammation and edema, and make surgery more difficult^2^. Patients with severe obstructive jaundice require preoperative biliary drainage^3^, which can be achieved through various methods, including ultrasound endoscopic biliary drainage (EUS-EBD) and endoscopic retrograde biliary drainage (ERBD). The latter is mainly divided into endoscopic nasobiliary drainage (ENBD) and endoscopic biliary stent (EBS). Some patients underwent PTBD when ERBD and EUS-EBD failed. The biliary infection rate under percutaneous transhepatic biliary drainage (PTBD) is significantly lower than that of ENBD and EBS^4,5^. US-guided PTBD represents the primary means of reducing jaundice in patients with DBMOJ. It is a valuable tool for preoperative jaundice reduction or palliative treatment. The aim of this study was to evaluate the clinical efficacy of US-guided PTBD in the treatment of different degrees of biliary dilatation caused by DBMOJ, as well as its success rate and complications.

## Methods

This study was approved by the Ethics Committee at our institution. Each patient signed a written informed consent form. It can be confirm that all procedures were performed in accordance with relevant guidelines and regulations. A retrospectively review of 89 patients with distal biliary malignant obstructive jaundice was conducted between July 2018 and July 2020. The number of males and females was 56 and 33, respectively. The median age of the patients was 59 years (range, 23–89y), and the diameter of the hepatic segment bile duct in the liver was 1.8–21.2 mm. The preoperative total bilirubin (TBIL), direct bilirubin (DBIL), alanine aminotransferase (AST) and aspartate aminotransferase (ALT) levels were 244.2 ± 55.7 μmol/L, 141.4 ± 36.7 μmol/L, 214.8 ± 56.1U/L and 144.2 ± 37.2U/L, respectively. Patients were grouped according to the diameter of the hepatic segment bile duct: group A with < 4mm, group B with 4–8 mm, and group C with > 8 mm. The corresponding bile duct drainage was then performed. The inclusion criteria for patients were as follows: Inclusion criteria: ① Clinical diagnosis of distal biliary malignant obstructive jaundice by computed tomography (CT) or magnetic resonance imaging(MRI); ② Preoperative imaging diagnosis showed intrahepatic and extrahepatic bile duct dilation; ③ No external biliary drainage; ④ No serious heart, liver and kidney failure and coagulation dysfunction. Exclusion criteria: Previous endoscopic retrograde cholangiography (ERCP) or ENBD with internal biliary drainage.

## Materials

The following devices were used for PTBD: Philip EPIQ5 ultrasound system (Netherlands), puncture frame and 18G needle (LEAPMED, China), 8F catheter (DIALL, China), 0.035 inches stiff guidewire.

### PTBD

**Figure 1:**
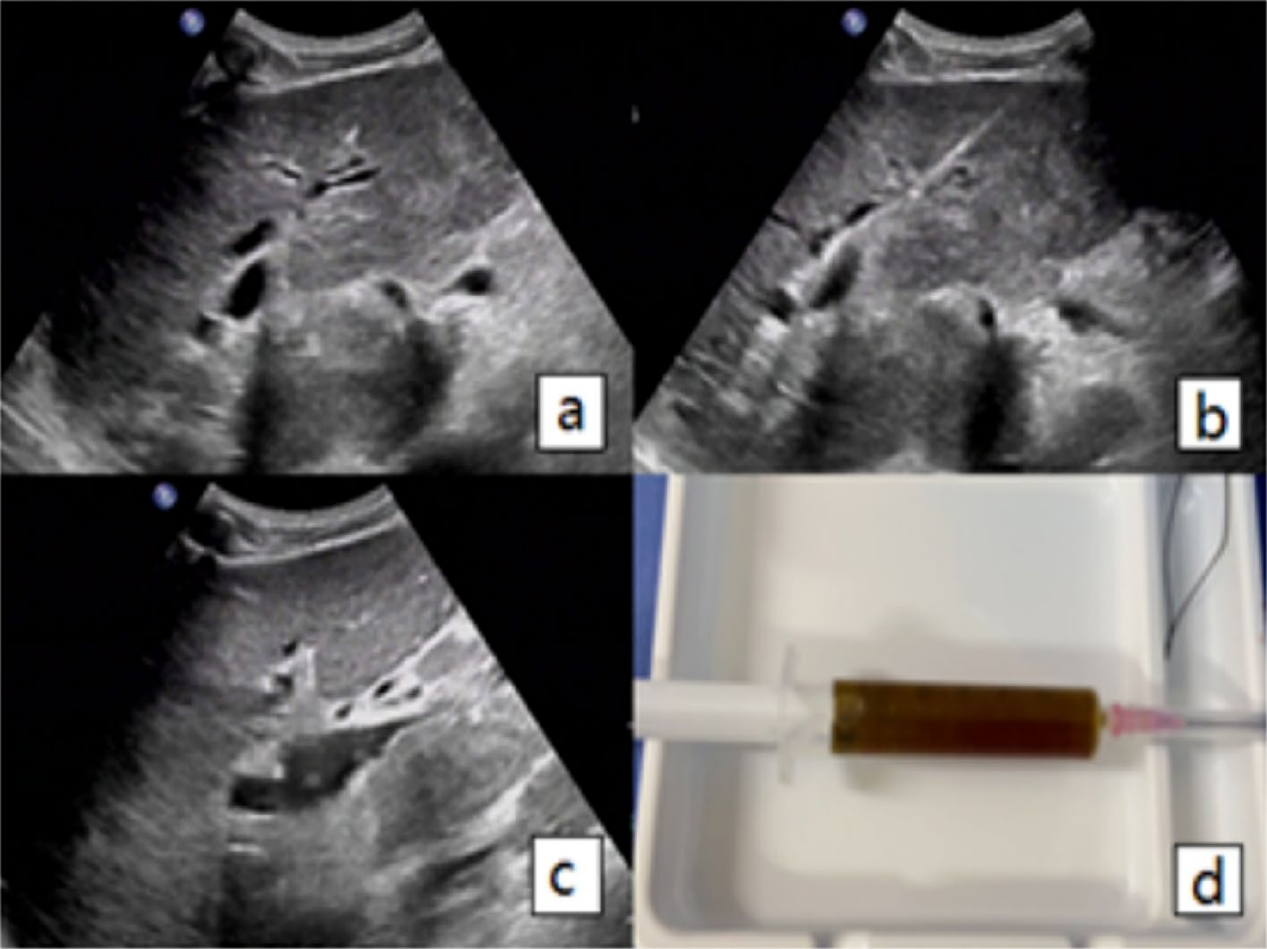
(**a**) Ultrasound image of a 67-year-old male patient with ampullary carcinoma who underwent PTBD, and the diameter of hepatic segment bile duct was 5mm. (**b**) Shows a guide wire being inserted into the hepatic S3 bile duct. (**c**) Shows an 8F drainage tube being inserted along the guide wire. (**d**) Shows the brown turbid bile fluid being drawn out.

Prior to the procedure, patients underwent a colour ultrasound examination to identify the dilated intrahepatic bile duct. This was followed by the determination of an appropriate needle position and access route. The puncture point and angle of the puncture needle were then adjusted according to the target bile tube. Following the application of a sterile protective cover and the administration of lidocaine anaesthesia to the liver envelope, the puncture needle was introduced into the expansive intrahepatic bile duct. Once the needle core had been removed, the bile flow was observed. A guide wire was then inserted and pushed through the intrahepatic bile duct with ultrasound guidance, after which an external drainage tube was fixed in place (Fig. 1).

### Clinical variables assessed

The following variables were recorded and compared between the three groups: the puncture success rates, complications, and liver function tests. The latter include TBIL, DBIL, ALT and AST.

### Statistical analysis

Categorical variables were summarized by frequencies and percentages and compared using Fisher exact tests or Pearson χ^2^ tests. Continuous variables were summarized by mean ± SD and compared using a two-sample independent t test. A statistical software package (SPSS, version 20.0; Chicago, IL) was used for data management and analysis. Two-tailed *P*-values below 0.05 were considered statistically significant.

### Ethical approval

Ethical approval for this study was obtained from the Ethics Committee of the Quanzhou First Hospital Affiliated to Fujian Medical University, and the patients consented to participate in the study.

## Results

### Patient characteristics

A total of 89 patients were included in the present study. Their median age was 59 years (range, 23–89 y), and the diameter of the hepatic segment bile duct in the liver was 1.8–21.2 mm. These characteristics and indications for PTBD are shown in Table 1. Their preoperative serum total bilirubin, direct bilirubin, alanine aminotransferase and aspartate aminotransferase were 244.2 ± 55.7μmol/L, 141.4 ± 36.7 μmol/L, 214.8 ± 56.1U/L and 144.2 ± 37.2U/L. The hepatic segment bile ducts with inner diameter < 4 mm were divided into group A, 4–8 mm was divided into group B, and > 8 mm was divided into group C, and the corresponding bile duct drainage was performed.

**Table 1:** The characteristics and indications of all patients.

	Group A	Group B	Group C
N	36	25	28
Age(y)	56 ± 7.6	58 ± 9.8	59 ± 11.3
Sex: male/female	20/16	17/8	19/9
Cause of obstruction			
Pancreatic carcinoma	8	8	7
Ampullary carcinoma	5	2	3
Cholangiocarcinoma	23	15	18
Diameter of the hepatic segment bile duct (mm)	3.4 ± 0.5	6.9 ± 0.7	12.6 ± 2.7

### The technical success rate

A total of 34 of the 36 cases in Group A, 25 of the 25 cases in Group B, and 28 of 28 cases in Group C were successful in the initial puncture. Following a repeat puncture, all of them were successful biliary drainage. There was no statistically significant difference between the three groups (*P* > 0.05).

### Complications

The early complications (bleeding, biliary fistula and bile reflex) and late complications (catheter comes off, catheter obstruction) of PTBD occurred in 2, 3, 3 cases and 2, 2, 3 cases in the three groups, respectively (Table 2).All complications were of grade 1 or 2 in the SIR Classification^6^. Bleeding occurred in 3.4% (n = 3) of the patients, none of which were severe, and all of which were in patients with left-side approach.

**Table 2:** Comparison of puncture success rate and complication rate among the three groups.

	Group A (36)	Group B (25)	Group C (28)	*P*
First puncture success rate	34 (94.4)	25 (100.0)	28 (100.0)	
Total puncture success rate	36 (100.0)	25 (100.0)	28 (100.0)	
Early complications (rate)	2 (5.6)	3 (12.0)	3 (10.7)	0.650
Bleeding	1	1	1	
Biliary fistula	1	1	2	
Biliary reflex	0	1	0	
Late complications (rate)	2 (5.6)	2 (8.0)	3 (10.7)	0.883
Drainage tube prolapsed	2	1	2	
Drainage tube obstruction	0	1	1	

### Liver function indicators

Postoperative 1 week, the TBIL percentage showed statistically significant differences among the three groups, while the DBIL percentage did not. The percentage difference between postoperative 1 week and 2 weeks of TBIL and DBIL was greater than that of ALT and AST.

**Figure 2:**
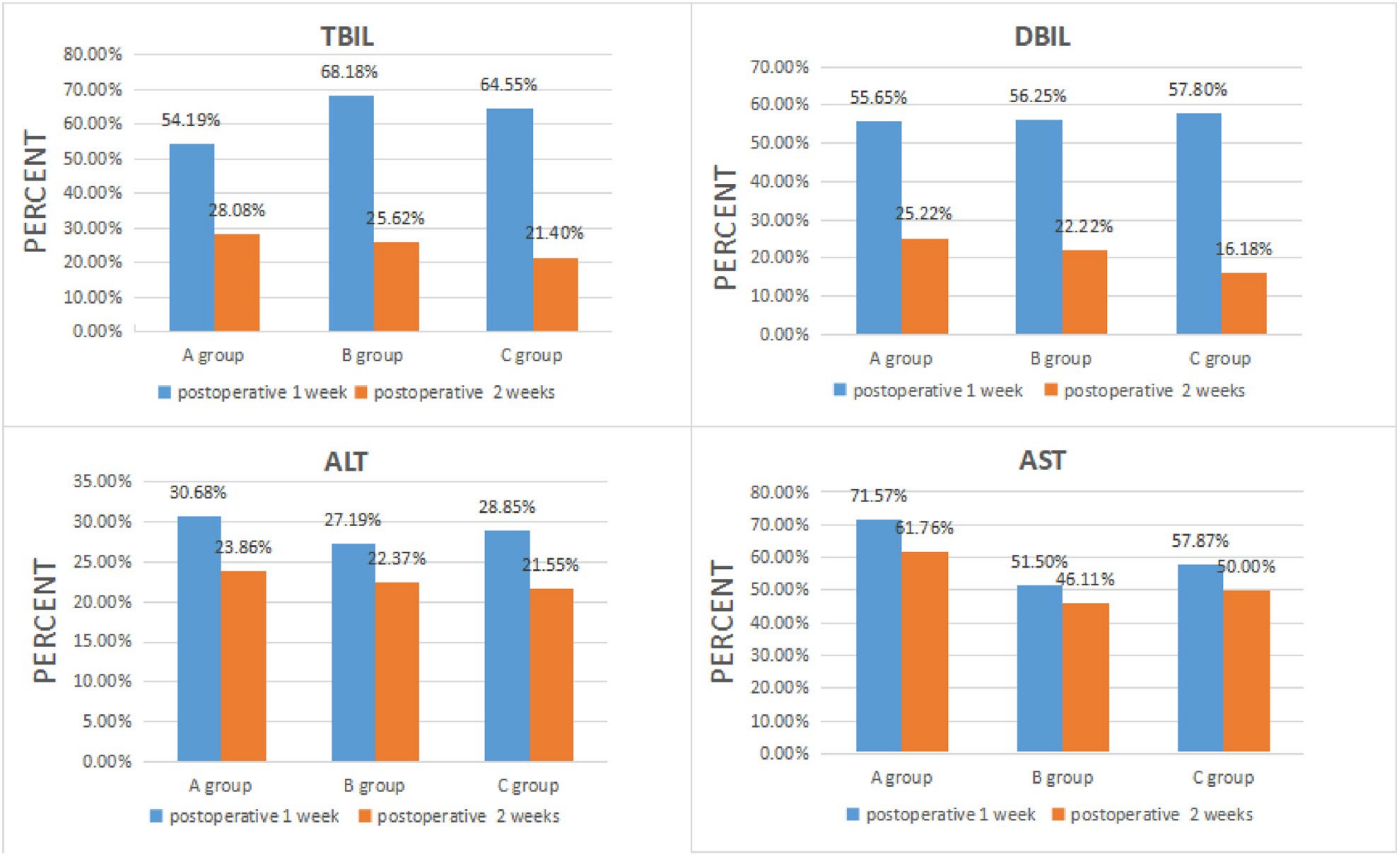
Trend chart of each liver function index.

ALT levels decreased to near-normal at postoperative 1 week, with no statistically significant differences observed among the three groups. Preoperative AST had the lowest value among the four measures. The AST at postoperative 1 and 2 weeks decreased to about half of the preoperation (Fig. 2).

The difference in AST between the three groups before operation was statistically significant, and the difference was not statistically significant after 2 weeks (Table 3).

**Table 3:** Comparison of liver function before and after PTBD in three groups.

	Preoperation				Postoperative 1 week				Postoperative 2 weeks			
	TBIL	DBIL	ALT	AST	TBIL	DBIL	ALT	AST	TBIL	DBIL	ALT	AST
Group A	203 ± 41	115 ± 24	176 ± 43	102 ± 31	110 ± 28	64 ± 19	54 ± 10	73 ± 17	57 ± 14	29 ± 17	42 ± 7	63 ± 15
Group B	242 ± 56	144 ± 40	228 ± 74	167 ± 85	165 ± 46	81 ± 23	62 ± 13	86 ± 27	62 ± 30	32 ± 13	51 ± 9	77 ± 17
Group C	299 ± 65	173 ± 41	253 ± 67	178 ± 103	193 ± 39	100 ± 26	73 ± 24	103 ± 35	64 ± 28	28 ± 11	57 ± 8	81 ± 20
F	2.017	2.110	1.569	4.593	3.343	1.343	1.658	1.096	0.103	0.093	2.411	1.176
P	0.146	0.134	0.221	0.016	0.050	0.278	0.209	0.348	0.903	0.912	0.112	0.326

*F: F value.

PTBD is divided into right- and left-side approaches. PTBD were performed through right access in 53 cases and through left access in 36 cases. The right-sided approach PTBD has a faster rate of liver function recovery in patients than the left-sided approach.

**Figure 3:**
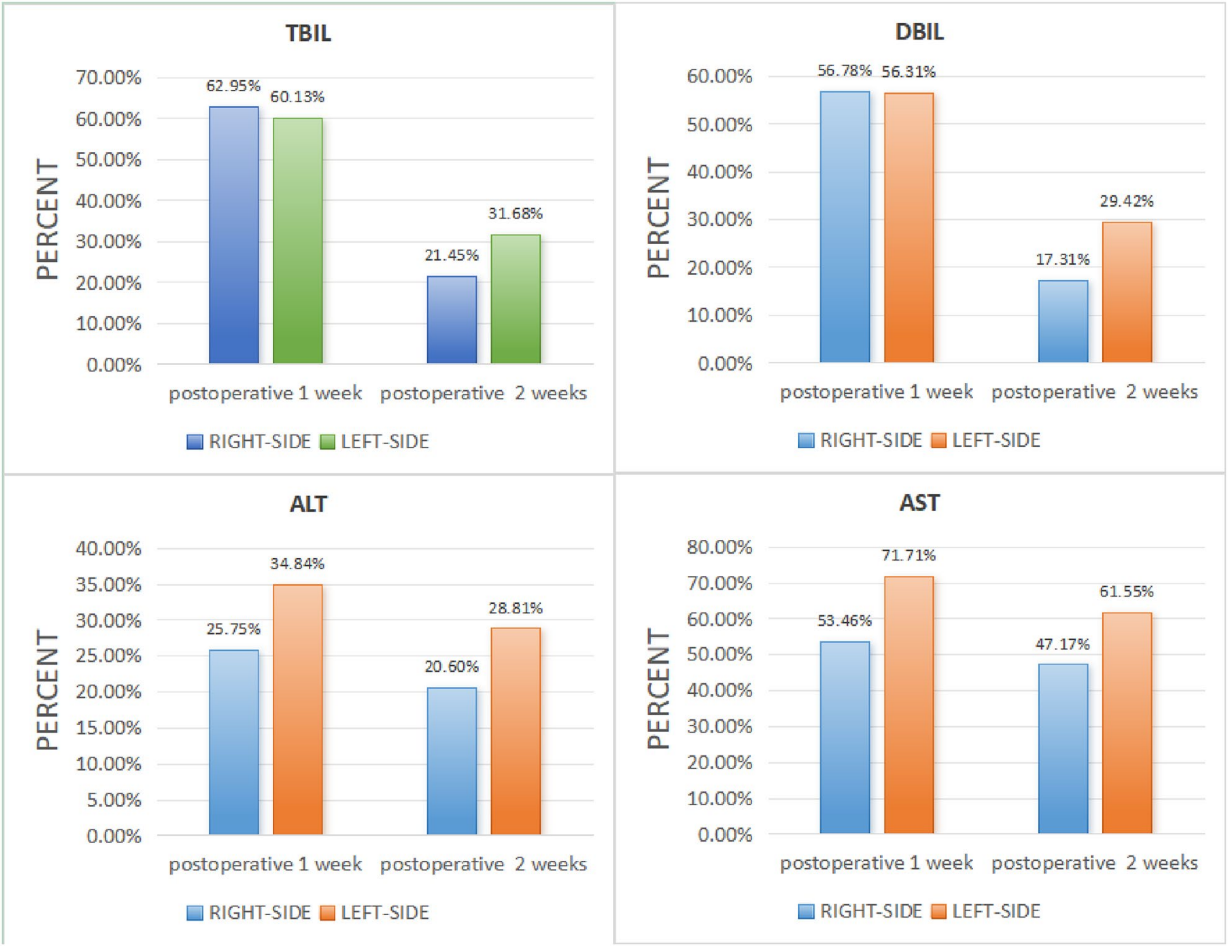
Relationship between puncture access and liver function.

Postoperative 1 week, there was no significant difference in TBIL and DBIL between right-side approach and left-side approach, while the difference was statistically significant after 2 weeks. Postoperative 1 week and 2 weeks, ALT and AST in right-side approach were significantly lower than those in left-side approach, and the difference was statistically significant (Fig. 3).

## Discussion

DBMOJ is mainly caused by stenosis or occlusion of the bile ducts, including pancreatic head cancer, ampullary cancer, cholangiocarcinoma and other malignant tumors. Bile excretion disorders, which result in cholestasis, can also lead to complications in the cardiovascular, nervous, and digestive systems^4^. This disease primarily affects middle-aged and elderly individuals, with most patients presenting to a doctor in the late stages of the disease. The clinical symptoms are severe, and most patients lose the opportunity for radical surgery. Timely and effective removal of biliary obstruction is crucial for reducing perioperative risks and postoperative complications, as well as improving the quality of life of patients with advanced tumors. The most common surgical procedures for jaundice reduction are endoscopic biliary drainage (EBD) and ERBD. Some patients underwent PTBD when ERBD and EUS-EBD failed. PTBD is further divided into PTBD and percutaneous transhepatic gallbladder drainage (PTGD) according to the puncture site^7^.

A meta-analysis has demonstrated that PTBD has a superior puncture success rate and a lower incidence of complications than EBD^8–10^. In EBD, a guide wire is retrogradely advanced through the obstruction under X-ray guidance through a digestive endoscopy, and a stent is placed. However, this approach is associated with a high risk of tumour bleeding at the blocked site and difficulty in controlling bleeding. In the event of a significant degree of stenosis or complete obstruction, this can result in catheter failure and a higher surgical conversion rate^11^. The incidence of complications is estimated to be approximately 30%, with the most common being tumour haemorrhage, acute pancreatitis, duodenal perforation, cholangitis and so on^10^.

US-guided PTBD allows for the clear visualisation of the needle path and needle tip position, thereby ensuring a safe and accurate procedure. However, complications may occur, with an incidence of approximately 25%, including biliary bleeding, pain, biliary fistula, and catheter obstruction^12^. Therefore, PTBD may be considered a clinical first choice for the reduction of jaundice. This study employs a prospective design to investigate the complications and treatment effects of PTBD on bile ducts with varying degrees of obstruction.

A previous study reported that PTGD has the characteristics of convenient operation, and has the same effect as PTBD in reducing jaundice^13^. PTGD is recommended for patients with a bile duct inner diameter of the bile duct less than 4 mm^14^. In our study, PTBD was performed on all patients improve the rigour of the experimental design. In group A, the second puncture was successful in 2 patients after the first puncture failed, and the overall puncture success rate reached 100%. In this study, haemorrhagic fluid drainage and abdominal effusion after drainage tube insertion were considered as bleeding. The differences in the incidence of early and late complications in the three groups were all relatively small and not statistically significant. Early complications are mainly due to abdominal pain caused by bile leakage during the puncture, which can be relieved by symptomatic treatment. Late complications are mild and the incidence is low, mainly due to prolapse and obstruction of the drainage tube^15,16^. In our study, 3.4% (n = 3) of patients experienced bleeding, none of which were severe, which was significantly lower than a previous study^17^. All three bleeding adverse events (AEs) occurred immediately after puncture. There was no evidence of extrahepatic bleeding. The drain was immediately clamped to increase the pressure on the drain to stop the bleeding. The study found that the occurrence of punctures, vascular punctures, fatty liver or cirrhosis and intrahepatic tumour obstruction are associated with haemorrhagic AEs. The S5 and S6 bile ducts are typically in an anterior position relative to the vessel, whereas the S3 bile duct is usually in the opposite position. Consequently, when performing PTBD for mild S3 bile duct dilatation, the needle must be turned to avoid the vessel, which typically requires a more dexterous puncture technique on the part of the physician. This contrasts with the results of a previous study^18^. Further study is required to investigate the puncture-related complications of the right and left hepatic approaches. However, it is recommended that even if the internal diameter of the intrahepatic bile duct is less than 4 mm, PTBD has better safety under the operation of high-level interventional physicians.

In our study, the clinical effects of drainage were evaluated quantitatively using bilirubin and transaminase indicators. The results showed that with the continuous external bile drainage, both bilirubin and transaminase gradually decreased. Preoperative AST was the lowest of the four measures.At 1 and 2 weeks postoperative, AST decreased to about half of the preoperation level. This suggests that patients with mild obstruction have a rapid recovery of TBIL after PTBD.

The present study aimed to investigate the impact of different approaches on liver function in patients with DBMOJ. The right-side approach PTBD has a faster rate of ALT and AST recovery than the left-side approach, and the puncture approach has no effect on the difference of TBIL and DBIL at 1 week postoperatively. This indicates that the right-side approach is better for biliary drainage than the left-side approach, and the drainage tube is unobstructed.

As the degree of obstruction increases, the cholestasis time is prolonged, and the ampullae of Hering, which connect the bile capillaries to the thin bile ducts, are mechanically ruptured. This results in direct bilirubin backflow into the blood and causing hyperbilirubinemia. Concurrently, the cytotoxic effect of bile acid results in the destruction of biofilm function and the development of hyperchloremia, which in turn causes damage to liver function and a continuous increase in transaminase levels^19^. As the obstruction is cleared, the pressure of the biliary tract decreases, and the reason for the initial recovery of transaminase may be attributed to the rapid decline in bile acid levels, the decline in toxicity, and the initial recovery of liver cell function. Consequently, the levels of ALT and AST decreased rapidly. However, the recovery from mechanical rupture of the ampulla of Hering is slower, and a small amount of bilirubin continues to flow back into the blood, resulting in a slower decline in bilirubin levels. This may be the reason why the rate of decline in bilirubin is slower than the rate of decline in transaminase.

There are some limitations to our study. The number of patients was relatively small, and the study was conducted at a single centre. The study has limitations, including a prolonged postoperative monitoring period and insufficient data to refine the changes in various liver function indicators.

## Conclusions

One week postoperatively, the level of AST dropped to the normal range, indicating that AST is a sensitive indicator of hepatocyte injury. PTBD for DBMOJ has the characteristics of a low complication rate and good efficacy in reducing jaundice. The lighter the obstruction, the faster the recovery of liver function. It is evident that liver function recovered more rapidly in patients with DBMOJ who underwent puncture through the right-sided approach in comparison to those who underwent puncture through the left-sided approach. It is therefore recommended that patients with DBMOJ should be treated with PTBD through the right-sided approach whenever feasible.

## Data Availability

All data generated or analysed during this study are included in this published article.
